# Maternal Dietary Strategies for Improving Offspring Cardiovascular–Kidney–Metabolic Health: A Scoping Review

**DOI:** 10.3390/ijms25189788

**Published:** 2024-09-10

**Authors:** You-Lin Tain, Chien-Ning Hsu

**Affiliations:** 1 Division of Pediatric Nephrology, Kaohsiung Chang Gung Memorial Hospital, Kaohsiung 833, Taiwan; tainyl@cgmh.org.tw; 2 College of Medicine, Chang Gung University, Taoyuan 333, Taiwan; 3 Institute for Translational Research in Biomedicine, Kaohsiung Chang Gung Memorial Hospital, Kaohsiung 833, Taiwan; 4 Department of Pharmacy, Kaohsiung Chang Gung Memorial Hospital, Kaohsiung 833, Taiwan; 5 School of Pharmacy, Kaohsiung Medical University, Kaohsiung 807, Taiwan

**Keywords:** metabolic syndrome, nutrition, cardiovascular disease, pregnancy, chronic kidney disease, diet, hypertension, developmental origins of health and disease (DOHaD)

## Abstract

Dietary regulation has been recognized for its profound impact on human health. The convergence of cardiovascular, kidney, and metabolic disorders at the pathophysiological level has given rise to cardiovascular–kidney–metabolic (CKM) syndrome, which constitutes a significant global health burden. Maternal dietary nutrients play a crucial role in fetal development, influencing various programmed processes. This review emphasizes the effects of different types of dietary interventions on each component of CKM syndrome in both preclinical and clinical settings. We also provide an overview of potential maternal dietary strategies, including amino acid supplementation, lipid-associated diets, micronutrients, gut microbiota-targeted diets, and plant polyphenols, aimed at preventing CKM syndrome in offspring. Additionally, we discuss the mechanisms mediated by nutrient-sensing signals that contribute to CKM programming. Altogether, we underscore the interaction between maternal dietary interventions and the risk of CKM syndrome in offspring, emphasizing the need for continued research to facilitate their clinical translation.

## 1. Introduction

Metabolic syndrome is a cluster of risk factors that significantly increases the likelihood of developing cardiovascular disease (CVD). Traditionally, metabolic syndrome focuses on a specific set of metabolic disturbances, including insulin resistance, obesity, and dyslipidemia. However, the American Heart Association has proposed a more comprehensive framework that incorporates a holistic view of health by integrating cardiovascular and kidney issues along with metabolic factors. This integrated approach results in the concept of cardiovascular–kidney–metabolic (CKM) syndrome [1]. CKM syndrome represents a clinical intersection where the interconnected nature of cardiovascular, kidney, and metabolic health is recognized and addressed. The complex interplay among these systems underscores the need for tailored interventions that address their interconnected nature.

Modern dietary patterns are closely linked to the high prevalence of metabolic syndrome, cardiovascular disease, and kidney disease [2]. Contemporary diets often include excessive calories, heavily processed foods, and high amounts of salt, trans fats, and added sugars. Significant changes in the frequency, quantity, and quality of dietary intake contribute to maladaptation and the development of various chronic diseases. Conversely, precise dietary management is crucial for managing these health conditions and extending lifespans [3].

Maternal dietary nutrients profoundly shape fetal development [4,5]. Disparities in maternal diets are linked to the onset of various adult-onset diseases, including each component of the CKM syndrome [6,7,8]. This concept is widely recognized as the developmental origins of health and disease (DOHaD) [9,10]. A notable example is the Dutch famine study, in which it was found that maternal undernutrition during pregnancy is linked to an increased risk of adult offspring developing coronary heart disease, hyperlipidemia, obesity, kidney disease, and hypertension—all features of CKM syndrome [11]. Furthermore, emerging evidence suggests that interventions during critical developmental stages can mitigate or even reverse the adverse effects associated with developmental programming, a process known as reprogramming [12]. This highlights the potential for regulating maternal diet to function as a reprogramming strategy for preventing disorders associated with DOHaD, including the CKM syndrome.

Thus, the objective of this review is to evaluate the influence of maternal diet regulation on offspring outcomes by synthesizing existing human and animal data, with a specific focus on CKM syndrome.

## 2. Results and Discussion

### 2.1. Dietary Intervention and CKM Syndrome

In light of the omnipresence of dietary nutrient-mediated signaling throughout the human body [13], it is recognized that diet-derived impacts on health and disease extend across all organ systems [14]. To date, several dietary interventions have been shown to improve the different components of CKM syndrome [2].

#### 2.1.1. Cardiovascular Disease

Diet is tightly connected to cardiovascular health [3,14]. For example, the Western diet is associated with an increased risk of CVD, while several other diets are linked to a reduced risk. These include the Mediterranean diet, the DASH diet (dietary approaches to stop hypertension), vegetarian diet, and plant-based diets.

Western diets and diets high in branched chain amino acids (BCAAs) are well-known risk factors for CVD [15,16]. Conversely, certain diet interventions are able to diminish adverse factors for CVD and improve cardiovascular outcomes [2]. Calorie restriction can enhance healthy aging. A 3-month fasting-mimicking diet (low in sugars, calories, and protein but high in unsaturated fats) has been reported to reduce bodyweight and total body fat, lower blood pressure (BP), and lessen dyslipidemia, all of which are risk factors for CVD [17]. Another study revealed that carbohydrate restriction in cases with type 2 diabetes (T2DM) improved most biomarkers of CVD risk after 1 year [18]. Studies have further demonstrated that several types of diets have a positive effect on reducing BP, including a vegetarian diet [19], the DASH diet [20], Mediterranean diet [21], and fermented/probiotic diet [22]. The DASH diet emphasizes the consumption of vegetables, fruits, legumes, nuts, whole grains, lean protein, and low-fat dairy products [20]. Similarly, the Mediterranean diet is distinguished by high intakes of grains, vegetables, fruits, fish, legumes, extra virgin olive oil, nuts, and the moderate consumption of red wine [21]. In particular, the Mediterranean diet can alter microbial composition and function to benefit cardiovascular health and prevent CVD [23,24].

#### 2.1.2. Chronic Kidney Disease

In advanced chronic kidney disease (CKD), patients frequently face protein–energy wasting due to multiple interrelated factors. Decreased appetite, often caused by metabolic alterations and systemic inflammation, impairs food intake. Additionally, gastrointestinal nutrient absorption is compromised, hindering the efficient processing and utilization of nutrients [25,26,27]. The accumulation of nitrogen-containing waste products, such as urea and creatinine, further aggravates this condition by inducing metabolic imbalances and reducing appetite. Coupled with increased muscle and fat wasting due to impaired protein synthesis and energy balance, these issues necessitate targeted dietary adjustments to support nutritional needs and overall health in this population.

Protein restriction is recommended in patients with CKD, as it retards the rate of renal function decline [28]. Likewise, the restriction of dietary sodium is recommended for the management of CKD and its associated risks [29]. As hyperkalemia and hyperphosphatemia are common complications, restricting dietary potassium and phosphorus intake is often recommended for patients with CKD [26]. Additional calcium and vitamin D supplementation may be offered to patients with advanced CKD. Furthermore, the DASH diet, plant-based diet, and the Mediterranean diet are currently being shown to play a potential role in delaying CKD progression. All together, these studies confirm that optimized diets play a pivotal role in alleviating CKD [30].

#### 2.1.3. Obesity and Diabetes

Overnutrition contributes to obesity and diabetes. Consuming a diet high in fats and excessive amounts of BCAAs, methionine, and tryptophan contribute to weight gain and obesity in rodent models, but human studies are limited [31]. Conversely, dietary interventions can be used to combat obesity-associated disorders. Calorie restriction, with or without time-restricted eating, showed similar bodyweight-lowering effects in patients with obesity [32]. The ketogenic diet is defined by its low-carbohydrate, high-fat, and normal protein composition, which triggers the production of ketone bodies by mimicking the breakdown of a fasting state. A meta-analysis revealed that the ketogenic diet had a positive effect on decreases in blood glucose, lipid control, and weight loss among patients with T2DM [33]. Additionally, plant-rich diets and the Mediterranean diet are associated with a lower risk of T2DM and obesity [34,35]. Moreover, daily high-fiber supplementation combined with fecal microbiota transplantation (FMT) alleviates insulin resistance in patients with obesity or metabolic syndrome [36].

#### 2.1.4. Dyslipidemia and Fatty Liver

Dyslipidemia has a decisive role in the development of non-alcoholic fatty liver disease (NAFLD), which is the result of metabolic disorders such as obesity, insulin resistance, and metabolic syndrome. The accumulation of free fatty acids and lipid metabolites within liver cells disturbs insulin signaling, resulting in the development of NAFLD [37]. Dietary cholesterol is a key factor in activating the inflammatory pathways underlying NAFLD [38], while regulating dietary composition may benefit both NAFLD and dyslipidemia. A dose–response relationship between the degree of calorie restriction and beneficial effects on liver function and weight loss was demonstrated [39]. A meta-analysis suggests that the Mediterranean diet may be an effective diet therapy for NAFLD [39]. Other plant-based diets, such as the DASH and vegetarian diets, are also beneficial, although more data are needed to establish the roles of the ketogenic diet and intermittent fasting in NAFLD [40].

### 2.2. Maternal Dietary Intervention for Offspring CKM Syndrome: Human Evidence

As elucidated in this review, there are clear correlations between diet regulation and the prevention of CKM syndrome. Maternal diets have a decisive role in fetal development, impacting the health and disease outcomes of offspring [41]. Dietary interventions started during pregnancy are essential to prevent downstream complications for both mothers and their children; however, evidence with respect to these interventions in humans, particularly concerning CKM syndrome, remains limited.

A healthy diet for a pregnant woman should consist of a diverse range of nutrient-rich whole foods, including vegetables, fruits, whole grains, and healthy fats with omega-3 fatty acids sourced from nuts, fish, and seeds [42]. It is advisable to prioritize these foods over less nutritious, heavily processed options. However, maternal nutritional requirements vary based on individual characteristics. In addition to assessing pre-pregnancy dietary quality, it is important to consider factors such as maternal body size, age, gestational age, activity level, multiple pregnancies, and medical conditions. Pregnant women should avoid diets that excessively restrict any macronutrient category. The Mediterranean diet, DASH diet, and the Nordic diet are considered optimal during pregnancy, while high-protein, low-carbohydrate diets, such as ketogenic and modified Atkins diets, should be avoided [42].

Special populations of pregnant women with distinct nutritional requirements include adolescent girls, and those with gestational diabetes mellitus (GDM), overweight/obesity, preeclampsia, and underweight conditions. Developing tailored strategies for these groups is highly recommended to meet the specific nutritional needs of each condition effectively. For example, nutrition therapy, based on carbohydrate restriction, is the foundation for the treatment of gestational diabetes mellitus (GDM). However, restricting dietary carbohydrates can lead to an increase in dietary fat intake. Accordingly, various dietary strategies for GDM have been reported, including low glycemic index (GI) diets, energy restriction, adjustments in carbohydrate levels, and modifications in the quality or quantity of fats or proteins. A meta-analysis of 18 randomized controlled trials (RCTs) and 8 dietary patterns for GDM nutrition revealed that improving nutritional quality and intake positively impacted outcomes related to maternal glycemia and birth weight [43].

Prior work indicates that fetal and newborn anthropometry was the most observed outcome related to maternal diets and child health [44]. Although some reports suggested that a mother’s adherence to Mediterranean dietary patterns was associated with a decreased risk of offspring obesity [45,46,47], there were inconsistent findings observed by others [48,49]. However, no evidence was reported regarding how maternal diet regulation protects offspring against other phenotypes of CKM syndrome.

### 2.3. Maternal Dietary Interventions as Reprogramming Strategies

In reprogramming strategies, the goal is to reverse or delay adverse programmed processes and foster normal development. Included in these strategies are nutritional interventions, lifestyle modifications, pharmacological therapy, and exercise. Given the limited information available from human studies, animal models with precise control over dietary regulation are crucial for discovering suitable maternal dietary interventions to prevent offspring CKM syndrome before implementing them in humans.

In this review, we were restricted to dietary interventions during gestation and breastfeeding periods as reprogramming strategies aiming at averting offspring CKM syndrome in all sorts of animal models [50,51,52,53,54,55,56,57,58,59,60,61,62,63,64,65,66,67,68,69,70,71,72,73,74,75,76,77,78,79,80,81,82,83,84,85,86,87,88,89,90,91,92,93,94,95,96,97,98,99,100,101,102,103,104,105], as detailed in Table 1, Table 2, Table 3, Table 4 and Table 5. Rats are the most commonly used species, followed by mice and rabbits. It should be noted that the developmental window is not uniform across different species and organ systems. In contrast to humans, kidney development in rodents progresses until approximately postnatal weeks 1–2 [106]. As a result, nutritional interventions during gestation and breastfeeding can help to preserve nephrogenesis and enhance nephron numbers, thereby mitigating kidney diseases originating from developmental factors [106].

Notably, dietary interventions employed for CKM syndrome may yield contrasting or potentially adverse outcomes on the developmental origins of CKM syndrome. For instance, dietary intervention with caloric restriction improves metabolic syndrome [107], kidney disease [108], and cardiovascular disease [109]. However, maternal caloric restriction has resulted in various components of CKM syndrome in adult rat offspring, including obesity, hypertension, kidney disease, hyperleptinemia, and hyperinsulinism [52,67,74,110]. Comparable adverse outcomes in offspring have been noted in both cows and ewes [111,112].

Dietary interventions during pregnancy and lactation are grouped into amino acid supplementation, lipid-associated diet, micronutrients, gut-microbiota-targeted diet, and plant polyphenols. Each will be discussed in detail in turn.

#### 2.3.1. Amino Acid Supplementation

Macronutrients utilized as dietary interventions primarily focus on amino acid supplementation. Amino acids serve as building blocks for a diverse array of structural proteins within the body, thereby playing crucial roles in organogenesis and fetal development. Among them, the most frequently utilized as reprogramming interventions are citrulline and taurine (Table 1).

Citrulline is a non-essential amino acid that can be metabolized into arginine. Both amino acids are purported to increase nitric oxide (NO) production and provide benefits for cardiovascular disease [113]. Supplementation with maternal citrulline has been shown to safeguard adult offspring from hypertension and kidney disease across various models of developmental programming, including, as follows: streptozotocin (STZ)-induced diabetes [51]; maternal caloric restriction [52]; co-administration of N^G^-nitro-L-arginine-methyl ester (L-NAME, NO synthase inhibitor) and dexamethasone [53]; maternal CKD [54]; antenatal dexamethasone exposure [55]; and spontaneously hypertensive rat (SHR, a genetic hypertensive rat model) [56]. While post-weaning arginine supplementation has been shown to enhance hypertension, insulin sensitivity, and beta cell function in adult rat offspring [114,115], the reprogramming effects of maternal arginine supplementation remain unexplored. Citrulline supplementation is more effective than arginine in generating NO because citrulline can bypass hepatic metabolism and undergo renal conversion to arginine [116]. Hence, before its implementation in humans, further exploration is warranted to enhance our understanding of how maternal citrulline supplementation can prevent developmental programming in various animal models.

Taurine is another amino acid commonly supplemented during gestation. As indicated in Table 1, perinatal use of taurine has been extensively studied in various components of CKM syndrome, including obesity, dyslipidemia, diabetes, hypertension, and kidney disease [57,58,59,60,61,62,63,64]. Taurine, the most plentiful sulfur-containing amino acid [117], is primarily obtained through dietary sources, although it can also be synthesized from cysteine. Maternal taurine supplementation can improve offspring obesity primed by maternal dyslipidemia [61] and a maternal high-fructose/high-fat diet [62]. Additionally, taurine supplementation perinatally protected offspring against hypertension in stroke-prone spontaneously hypertensive rats (SHRSP) and SHRs [59,63]. Using a non-obese diabetic (NOD) mice model, the use of taurine during pregnancy and lactation delayed the onset time of diabetes from 30 to 38 weeks in male offspring and from 18 to 30 weeks in female offspring [64]. In maternal CKD, the protective actions of perinatal taurine supplementation on offspring hypertension and kidney disease are closely associated with the modulation of gut microbiota [58]. Taurine supplementation led to an increased presence of *Dehalobacterium*, *Bifidobacterium*, and *Asteroleplasma* genera, alongside a decrease in *Erisipelactoclostridium* [58]. The replenishment of *Bifidobacterium* levels, which had declined due to maternal CKD, was linked to taurine’s probiotic properties, which contribute towards preventing hypertension [58]. Using the same model, gestational supplements with cysteine or tryptophan also showed beneficial effects on offspring hypertension complicated by maternal CKD [64,65]. Glycine is a simple amino acid that is not essential in the human diet. In a rat model, glycine supplementation during gestation averted hypertension in progeny born to dams that experienced protein restriction [50]. Moreover, maternal BCAA supplementation has been shown to protect adult rat offspring against hypertension, obesity, and diabetes in maternal caloric restriction and high-fat diet models, respectively [67,68].

**Table 1:** Effects of maternal amino acid supplementation on offspring CKM phenotypes in animal models.

Dietary Intervention	Dose	Periods Pregnancy/Lactation	Model	Species	Prevented CKM Phenotypes	Age at Measure (Weeks)	Ref.
Glycine	3%	Yes/No	Protein restriction	Rat	Hypertension	4	[50]
Citrulline	0.25%	Yes/Yes	STZ-induced diabetes	Rat	Hypertension and kidney disease	12	[51]
Citrulline	0.25%	Yes/Yes	Caloric restriction	Rat	Kidney disease	12	[52]
Citrulline	0.25%	Yes/Yes	Maternal L-NAME exposure	Rat	Hypertension	12	[53]
Citrulline	0.25%	Yes/Yes	Maternal CKD	Rat	Hypertension	12	[54]
Citrulline	0.25%	Yes/Yes	Prenatal dexamethasone exposure	Rat	Hypertension and kidney disease	16	[55]
Citrulline	0.25%	Yes/Yes	SHR	Rat	Hypertension	50	[56]
Taurine	3%	Yes/Yes	Maternal high sugar diet	Rat	Hypertension	8	[57]
Taurine	3%	Yes/Yes	Maternal CKD	Rat	Hypertension and kidney disease	12	[58]
Taurine	3%	Yes/Yes	SHRSP	Rat	Hypertension	12	[59]
Taurine	3%	Yes/Yes	STZ-induced diabetes	Rat	Hypertension	16	[60]
Taurine	3%	Yes/Yes	Maternal dyslipidemia	Rat	Obesity, dyslipidemia, and hypertension	16	[61]
Taurine	1.5%	Yes/Yes	Maternal high-fructose/high-fat diet	Rat	Obesity	21	[62]
Taurine	3%	Yes/Yes	SHR	Rat	Hypertension	22	[63]
Taurine	2.5%	Yes/Yes	NOD	Mouse	Diabetes	50	[64]
Cysteine	8 mmol/kg/day	Yes/No	Maternal CKD	Rat	Hypertension	12	[65]
Tryptophan	200 mg/kg BW/day	Yes/No	Maternal CKD	Rat	Hypertension	12	[66]
BCAAs	NA	Yes/No	Caloric restriction	Rat	Hypertension	16	[67]
Leucine	1.5% chow	Yes/Yes	High-fat diet	Mouse	Obesity and diabetes	16	[68]

BCAA, branched chain amino acid; STZ, streptozotocin; L-NAME, N^G^-nitro–L-arginine methyl ester; CKD, chronic kidney disease; SHR, spontaneously hypertensive rat; SHRSP, stroke-prone spontaneously hypertensive rat; NOD, non-obese diabetic; NA, not available.

#### 2.3.2. Lipid-Associated Diet

Dietary fat, the most caloric-rich macronutrient, breaks down into fatty acids that perform essential physiological functions. Generally, the consumption of saturated fatty acids and trans fats is associated with a heightened risk of CVD. In contrast, monounsaturated and polyunsaturated fatty acids (PUFAs) are associated with a decreased risk of CVD [118]. As shown in Table 2, maternal consumption diets high in saturated fats resulted in obesity, diabetes, hypertension, dyslipidemia, and fatty liver in adult offspring [68,69,77,78,81]. Conversely, PUFA supplementation has been used in reprogramming interventions against offspring CKM syndrome. To date, three reports have demonstrated that PUFA supplementation during gestation and lactation has beneficial effects against hypertension [69,70], CVD [70], and fatty liver [71] in adult rat offspring. Conjugated linoleic acid (CLA) primarily originates from dietary PUFAs, especially linoleic acid, which is present in various foods. Supplementation with CLA during gestation and breastfeeding has been shown to protect adult rat offspring from hypertension induced by a high-fat diet [69]. Despite recommendations for pregnant and breastfeeding women to consume PUFAs [119], a meta-analysis involving 3644 children revealed that maternal supplementation with omega-3 PUFAs during pregnancy does not significantly impact obesity risk [120]. Consequently, the potential beneficial or detrimental effects of individual PUFAs used as dietary supplements during gestation on the offspring’s risk of CKM syndrome remain undecided.

**Table 2:** Effects of maternal lipid-associated diet on offspring CKM phenotypes in animal models.

Dietary Intervention	Dose	Periods Pregnancy/Lactation	Model	Species	Prevented CKM Phenotypes	Age at Measure (Weeks)	Ref.
Lipid-associated diet							
Conjugated linoleic acid	1% chow	Yes/Yes	Maternal high-fat diet	Rat	Hypertension	18	[69]
PUFA	1.5 g/kg/day	Yes/Yes	Protein restriction	Rat	Hypertension and cardiovascular disease	24	[70]
PUFA	8.78% chow	Yes/Yes	Maternal cafeteria diet	Rat	Fatty liver	56	[71]

PUFA, polyunsaturated fatty acids.

#### 2.3.3. Micronutrients

Micronutrients consist of vitamins and minerals. Vitamins C and E, as well as selenium, etc., have antioxidant properties and exhibit advantageous effects on human health [121]. Among the antioxidant supplements, vitamins C and E are the most commonly utilized. As a water-soluble antioxidant, vitamin C acts as a scavenger of free radicals and serves as a reducing agent [122]. On the other hand, vitamin E, being lipid-soluble, works by inhibiting various oxidative enzymes, thereby decreasing ROS production [123]. As shown in Table 3, supplementation with either vitamin C or E alone during pregnancy protected against offspring hypertension induced by maternal lipopolysaccharide (LPS) exposure [72,73]. Additionally, vitamin C and E, in combination with selenium and folic acid, protected against hypertension and CVD in adult rat offspring born to dams experiencing caloric restriction [74]. A causal link between maternal hypercholesterolemia and the development of atherosclerosis later in life has been established in rabbits [124]. Using this rabbit model, treatment with vitamin E demonstrated protective effects against the progression of atherosclerosis in adult rabbit offspring [75].

An essential water-soluble B vitamin, folic acid is widely present in various fruits and vegetables. It, along with choline and betaine, serves as a source of the coenzymes involved in one-carbon metabolism [125]. One-carbon metabolites work as methyl donors that are required for DNA methylation. One study showed that gestational supplementation with folic acid protected against hypertension and CVD in adult rat progeny born to dams experiencing protein restriction [76]. Feeding mice a high-fat diet and supplementing with choline before mating and during gestation had a protective effect on the development of obesity and fatty liver in offspring maintained on a high-fat diet [77,78]. Another report showed that maternal betaine supplementation attenuated dyslipidemia and fatty liver in adult rat offspring exposed to dexamethasone [79]. Although supplementation with folic acid and other methyl donors during gestation has been recommended to improve certain offspring outcomes [126], a diet high in folic acid or methyl donors may increase the offspring’s susceptibility to negative health outcomes later in life, including hypertension, hyperlipidemia, and insulin resistance [127,128]. It is important to note that vitamin supplements should be given only when there is a documented deficiency and not as a routine practice during pregnancy.

**Table 3:** Effects of maternal micronutrient supplementation on offspring CKM phenotypes in animal models.

Dietary Intervention	Dose	Periods Pregnany/Lactation	Model	Species	Prevented CKM Phenotypes	Age at Measure (Weeks)	Ref.
Vitamin C	350 mg/kg/day	Yes/No	Prenatal LPS exposure	Rat	Hypertension	12	[72]
Vitamin E	350 mg/kg/day	Yes/No	Prenatal LPS exposure	Rat	Hypertension and kidney disease	17	[73]
Vitamin C, E, selenium and folic acid	Combined doses ^1^	Yes/No	Caloric restriction	Rat	Cardiovascular disease and hypertension	16	[74]
Vitamin E	350 mg/kg/day	Yes/No	Cholesterol-enriched diet	Rabbit	Cardiovascular disease and hypertension	24	[75]
Folic acid	5 mg/kg/day	Yes/No	Protein restriction	Rat	Cardiovascular disease and hypertension	15	[76]
Choline	11.7 mmol/kg in chow	Yes/No	High-fat diet	Mouse	Obesity	9	[77]
Choline	25 mM in water	Yes/No	High-fat diet	Mouse	Fatty liver	9	[78]
Betaine	0.1 mg/kg/day i.p.	Yes/No	Postnatal dexamethasone exposure	Rat	Dyslipidemia and fatty liver	16	[79]

LPS, lipopolysaccharide. ^1^ alpha-tocopherol (250 mg/kg/day), ascorbic acid (150 mg/kg/day), selenium (0.3 mg/kg/day) and folic acid (4 mg/kg/day).

#### 2.3.4. Gut Microbiota-Targeted Diet

Consuming diets abundant in plant-based ingredients, fermented foods, and high-fiber foods is associated with a more diverse and beneficial gut microbiota. These dietary patterns significantly enhance cardiovascular–kidney–metabolic health by shaping gut microbiota and the derived metabolites [129]. Similarly, the Mediterranean diet, DASH diet, and a vegetarian diet can also nourish beneficial gut bacteria [130,131,132]. Accordingly, a gut-microbiota-targeted diet, which includes probiotics, prebiotics, and postbiotics, has emerged as a reprogramming strategy to avert CKM syndrome with developmental origins (Table 4).

Probiotics and prebiotics are often discussed and implemented in clinical practice. Probiotic therapy entails the intentional introduction of beneficial microorganisms into the gut microbiota [133]. Food ingredients that promote the growth or enhance the activity of beneficial microbes are referred to as prebiotics [134]. Metabolites produced by probiotics after processing, known as postbiotics, include vitamins, secreted proteins, short-chain fatty acids (SCFAs), and secreted biosurfactants [135].

*Lactobacillus casei* and *Lactiplantibacillus plantarum WJL*, both probiotics, demonstrated reprogramming effects by enhancing gut microbiota diversity and mitigating conditions like hypertension, dyslipidemia, and insulin resistance [80,81,82,83]. Similarly, prebiotics—including inulin, oligofructose, inositol, fructooligosaccharide, and garlic oil—have been effective in protecting against high-fat diet-induced hypertension, fatty liver, obesity, and diabetes in adult offspring [80,81,82,83,84,85,86,87]. Moreover, a high-fiber diet has also been utilized as a reprogramming strategy to avert the developmental programming of obesity and diabetes [95,96].

Many foods demonstrate prebiotic activity by enhancing the growth of beneficial microbes in the gut. Abundant in polysulfides, garlic (*Allium sativum*) serves as a dietary source of hydrogen sulfide (H_2_S) donors [136]. This characteristic underpins its diverse health-promoting properties, which include cardiovascular protection, BP reduction, anti-inflammatory and antioxidant activities, prebiotic effects, and blood sugar regulation. Studies have shown that maternal supplementation with garlic oil can positively impact offspring predisposed to hypertension due to a high-fat diet. This supplementation has been associated with an increased abundance of beneficial microbes such as *Bifidobacterium* and *Lactobacillus*; higher levels of acetic acid, butyric acid, and propionic acid in plasma; and enhanced α-diversity.

SCFAs are key microbial metabolites that can function as postbiotics. Acetic acid, a plentiful SCFA, interacts with its receptors to regulate BP [137]. Previous research demonstrated that perinatal acetic acid supplementation could prevent hypertension in offspring programmed by a maternal high-fructose diet [88] or maternal minocycline exposure [89]. Another SCFA under investigation for reprogramming for CKM programming is propionic acid. Studies have revealed that propionic acid supplementation during gestation and lactation can protect adult offspring from diabetes, hypertension, and dyslipidemia [88,89,90,91]. Additionally, as a postbiotic, maternal butyric acid supplementation reversed hypertension in adult rat offspring born to dams fed a high-fructose diet [92] or a tryptophan-free diet [93]. Furthermore, butyric acid use during gestation and lactation improved diabetes outcomes in the adult offspring of nonobese diabetic mice [94].

**Table 4:** Effects of maternal gut microbiota-targeted diet on offspring CKM phenotypes in animal models.

Dietary Intervention	Dose	Periods Pregnancy/Lactation	Model	Species	Prevented CKM Phenotypes	Age at Measure (Weeks)	Ref.
*Lactobacillus casei*	2 × 10^8^ CFU/day	Yes/Yes	Maternal high-fructose diet	Rat	Hypertension	12	[80]
*Lactobacillus casei*	2 × 10^8^ CFU/day	Yes/Yes	High-fat diet	Rat	Hypertension	16	[81]
*Lactiplantibacillus plantarum WJL*	1 × 10^9^ CFU/day	Yes/Yes	Maternal high-fat/high-cholesterol diet	Rat	Hypertension, diabetes, and dyslipidemia	13	[82]
Multi-strain probiotics	Combined ^1^	Yes/Yes	Maternal high-fat diet	Mouse	Diabetes	20	[83]
Long-chain inulin	5% *w*/*w*	Yes/Yes	Maternal high-fructose diet	Rat	Hypertension	12	[80]
Long-chain inulin	5% *w*/*w*	Yes/Yes	High-fat diet	Rat	Hypertension	16	[81]
Oligofructose	10% *w*/*w*	Yes/Yes	Maternal high-fat/high-sucrose diet	Rat	Diabetes and fatty liver	24	[84]
Inositols	Myo-inositol/D-chiro-inositol: 7.2/0.18 mg/mL water	Yes/No	Maternal high-fat diet	Mouse	Hypertension and diabetes	10	[85]
Fructooligosaccharides	10% *w*/*w*	Yes/No	Maternal high-fat diet	Mice	Obesity and diabetes	12	[86]
Garlic oil	100 mg/kg/day	Yes/Yes	High-fat diet	Rat	Hypertension	16	[87]
Acetic acid	200 mmol/L	Yes/Yes	Maternal high-fructose diet	Rat	Hypertension	12	[88]
Acetic acid	200 mmol/L	Yes/Yes	Maternal minocycline administration	Rat	Hypertension	12	[89]
Propionic acid	200 mmol/L	Yes/Yes	Maternal high-fructose diet	Rat	Hypertension	12	[88]
Propionic acid	200 mmol/L	Yes/Yes	Maternal CKD	Rat	Hypertension	12	[90]
Propionic acid	200 mmol/L	Yes/Yes	Maternal hypoxia	Mouse	Diabetes and dyslipidemia	11	[91]
Butyric acid	400 mg/kg/day	Yes/Yes	Maternal high-fructose diet	Rat	Hypertension	12	[92]
Butyric acid	400 mg/kg/day	Yes/Yes	Maternal tryptophan-free diet	Rat	Hypertension	16	[93]
Butyric acid	400 mg/kg/day	Yes/Yes	NOD	Mouse	Diabetes	16	[94]
High-fiber diet	22% chow	Yes/Yes	Maternal diabetogenic diet	Rat	Diabetes	40	[95]
High-fiber diet	NA	Yes/Yes	Maternal Western diet	Mouse	Obesity and diabetes	8	[96]

CKD, chronic kidney disease; NOD, non-obese diabetic; NA, not available. ^1^ *B. breve* DM8310, *L. acidophilus* DM8302, *L. casei* DM8121 and *S. thermophilus* DM8309.

#### 2.3.5. Plant Polyphenols

Most plant-based diets are rich in polyphenols, the predominant group of phytochemicals, which are natural compounds synthesized exclusively by plants [138]. These include flavanones, flavanols, isoflavones, flavones, anthocyanins, stilbenes, xanthones, lignans, and tannins [138]. Dietary polyphenols help prevent certain diseases through various mechanisms such as antioxidant activity, prebiotic effects, and epigenetic modifications [139,140]. Furthermore, polyphenols exhibit reprogramming properties and are of significant interest in disease prevention research within the DOHaD framework [141].

Resveratrol, a natural polyphenol found in grapes, is generally recognized for its antioxidant, anti-inflammatory, and prebiotic properties [142]. It has been proposed as a preventive strategy to improve cardiometabolic health [143]. Studies listed in Table 5 showed that resveratrol has beneficial actions against offspring CKM syndrome, addressing issues such as hypertension, dyslipidemia, obesity, and fatty liver [97,98,99,100].

**Table 5:** Effects of maternal polyphenol supplementation on offspring CKM phenotypes in animal models.

Dietary Intervention	Dose	Periods Pregnancy/Lactation	Model	Species	Prevented CKM Phenotypes	Age at Measure (Weeks)	Ref.
Resveratrol	50 mg/L	Yes/Yes	Maternal CKD	Rat	Hypertension	12	[97]
Resveratrol	147 mg/kg/day	Yes/Yes	Maternal high-fat/sucrose diet	Rat	Diabetes and obesity	15	[98]
Resveratrol	50 mg/L	Yes/Yes	High-fat diet	Rat	Dyslipidemia, obesity, and fatty liver	16	[99]
Resveratrol	0.2% *w*/*w*	Yes/Yes	Maternal high-fat diet	Mouse	Dyslipidemia and obesity	14	[100]
Epigallocatechin gallate	0.1%	Yes/No	Prenatal dexamethasone exposure	Rat	Hypertension	14	[101]
Quercetin	50 mg/kg/day	Yes/No	Maternal high-fat diet	Mouse	Hypertension	24	[102]
Grape skin extract	200 mg/kg/day	No/Yes	Maternal high-fat diet	Rat	Hypertension	24	[103]
Curcumin	400 mg/kg/day	Yes/Yes	Maternal hyperglycemic diet	Mouse	Obesity, diabetes, and dyslipidemia	12	[104]
Green tea	0.24%	No/Yes	Protein restriction plus post-weaning high-fat diet	Rat	Kidney disease	45	[105]

CKD, chronic kidney disease.

**Figure 1:**
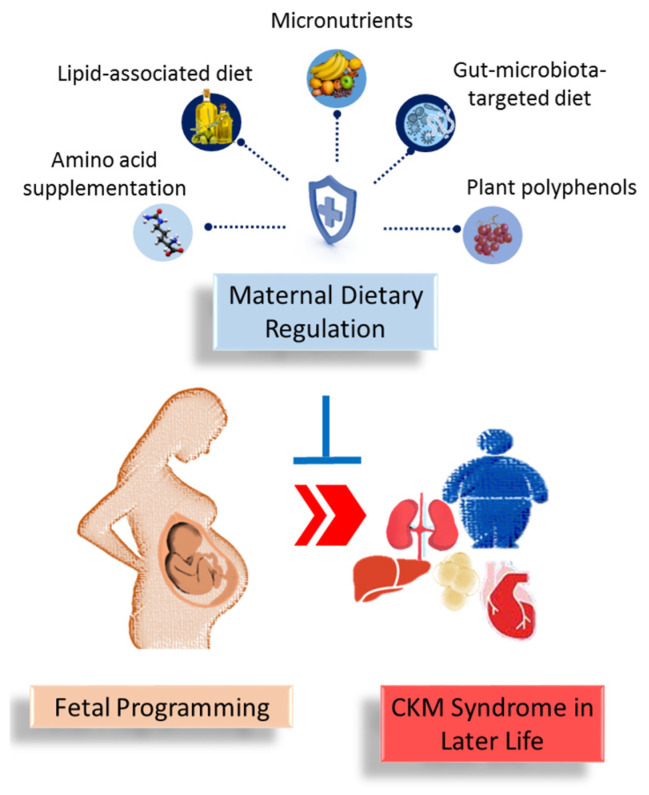
A summary of the role of maternal dietary regulation in preventing the developmental programming of cardiovascular–kidney–metabolic (CKM) syndrome in offspring later in life.

Another example of polyphenol flavonoids used for reprogramming in CKM programming is epigallocatechin gallate [86]. Perinatal use of epigallocatechin gallate attenuated offspring hypertension induced by antenatal dexamethasone exposure [101]. Quercetin, a polyphenol from the flavanols family, has demonstrated protective effects against offspring hypertension complicated by high-fat diets [102]. Additionally, grape skin extract, containing about 30% of total polyphenols, protected adult rat progeny from hypertension induced by maternal high-fat intake [103]. Furthermore, maternal supplementation with curcumin, a polyphenol found in turmeric, has been shown to reverse obesity, diabetes, and dyslipidemia in adult offspring primed by a maternal hyperglycemic diet [104]. Moreover, maternal intake of green tea polyphenols during lactation attenuated kidney disease in male offspring fed a high-fat diet and programmed by maternal protein restriction in rats [105]. As outlined in this review, Figure 1 illustrates the intricate associations between maternal dietary regulation, fetal programming, and offspring CKM syndrome.

### 2.4. Nutrient-Sensing Signals and CKM Programming

As mentioned above, maternal dietary intervention involves the strategic intake of various nutrients such as carbohydrates, amino acids, lipids, micronutrients, and metabolites. These nutrients trigger sensing signals that activate multiple biochemical pathways under different dietary conditions. As a result, the identification of specific molecular mechanisms underlying CKM programming and the targeting of these signaling pathways to develop ideal reprogramming interventions could offer new therapeutic opportunities. These nutrient-sensing signals include, as follows: AMP-activated protein kinase (AMPK); sirtuin (SIRT); peroxisome proliferator-activated receptors (PPARs); and PPARγ coactivator-1α (PGC-1α) [13]. To date, interventions during early life that target AMPK or PPAR signaling pathways have been documented to prevent CKM characteristics across various developmental programming models [144,145,146].

#### 2.4.1. AMPK

Maintaining cellular metabolism within a precise range requires tight regulation of ATP levels. AMPK, a universally expressed serine/threonine protein kinase with catalytic α subunits and regulatory β and γ subunits, plays a crucial role in this regulation [147]. The activation of AMPK occurs when cellular energy levels decrease, as indicated by elevated AMP-to-ATP or ADP-to-ATP ratios. Its primary function is to restore energy balance by enhancing energy production. Polyphenol-rich foods can act as indirect AMPK activators [148]. Certain indirect AMPK activators have revealed beneficial actions on programmed hypertension, including garlic [87], resveratrol [97], epigallocatechin gallate [101], and quercetin [102]. Additionally, the activation of AMPK-PGC1α-activity protected adult rat offspring from maternal western-style-diet-induced increased adiposity and fatty liver in later life [149].

#### 2.4.2. PPAR

Emerging evidence suggests that PPARs play a crucial role in the development of different aspects of CKM syndrome; interventions that activate them hold promise for treating these CKM-related conditions [145,150,151,152].

Nevertheless, only a few studies have assessed the influence of dietary PPAR modulators on CKM programming [145]. Certain natural PPAR agonists, such as omega-3 PUFAs and conjugated linoleic acid, have been studied in the developmental programming of hypertension, CVD, and fatty liver [69,70,71]. Due to the broad spectrum of affinity that fatty acid derivatives exhibit towards PPARs [153], determining whether their reprogramming effects are PPAR-dependent can be challenging. Another study showed that 15-Deoxy-Δ^12,14^-prostagandin J_2_ (15dPGJ_2_) treatment, a natural PPARγ ligand, averted programmed hypertension in maternal fructose-fed male adult rat offspring [154].

#### 2.4.3. Sirtuin

The SIRT family comprises seven proteins (SIRT1–SIRT7) categorized as class III histone deacetylases (HDACs) [155]. These enzymes require NAD^+^ as a cofactor and play a crucial role in epigenetic regulation, which is fundamental to developmental programming [156]. Specifically, SIRT1 mediates the deacetylation of PGC-1α, thereby influencing the expression of PPAR target genes. Reduced renal expression of SIRT1 has been associated with hypertension in offspring complicated by a maternal methyl-deficient diet [157]. Similarly, decreased SIRT4 expression in the kidney has been linked to hypertension complicated by maternal high-fructose diet [80].

Conversely, activation of the SIRT1-AMPK α-eNOS pathway has been shown to confer benefits in alleviating hypertension [158]. The overexpression of SIRT1 was reported to reduce BP in Ang II-induced hypertension, and this action was reversed by the SIRT1 inhibitor [159]. Several polyphenols, such as resveratrol, epigallocatechin gallate, quercetin, and curcumin, are known to activate SIRTs [160]. However, the extent to which the reprogramming effects of these polyphenols, as listed in Table 1, are mediated through SIRT activation and the specific concentrations required for these effects, are unclear.

#### 2.4.4. Others

Given that diverse maternal nutritional factors can lead to similar CKM phenotypes in adult offspring, there may be underlying mechanisms beyond nutrient-sensing signals that contribute to the pathogenesis of nutritional programming associated with CKM syndrome. Current research points to several potential mechanisms, including, as follows: oxidative stress [161,162]; epigenetic regulation [163]; gut microbiota [164]; inflammation [165]; and sex differences [166]. However, the exact effects of these mechanisms on maternal dietary interventions and their influence on the risk of CKM syndrome in offspring are not fully understood and require further investigation.

## 3. Materials and Methods

### 3.1. Search Strategy and Data Sources

**Figure 2:**
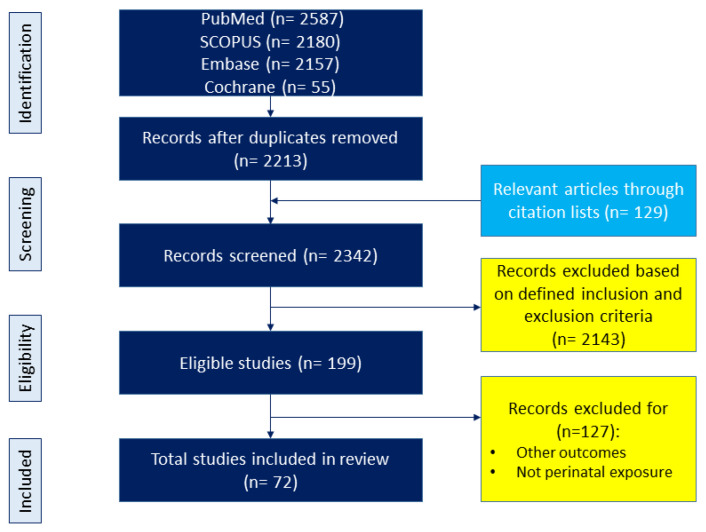
Flowchart of the literature search and selection.

The topic was adhered to maternal diet and its relation to offspring outcomes, with a focus on CKM phenotypes. We adhered to the preferred reporting items for PRISMA Extension for Scoping Reviews (PRISMA-ScR) guidelines throughout our review process. The diet variable included nutritional content, food-based interventions, dietary pattern and quality, and other dietary-related variables; however, it did not include maternal nutritional status and non-food-based interventions. Offspring outcomes were considered as all components of CKM syndrome starting from birth. The search covered keywords and their combinations such as “developmental programming”, “DOHaD”, “offspring”, “pregnancy”, “gestation”, “lactation”, “mother”, “progeny”, “reprogramming”, “diet”, “nutrition”, “carbohydrate”, “amino acid”, “fat”, “fiber”, “micronutrient,” “protein,” “fatty acid”, “food”, “metabolic syndrome”, “hypertension”, “diabetes”, “chronic kidney disease”, “fatty liver”, “obesity”, “hyperlipidemia”, and “cardiovascular disease”. Further selections were conducted in the article identification process. The study selection process is illustrated in Figure 2.

### 3.2. Article Identification

We conducted a search through scientific databases such as PubMed, SCOPUS, Embase, and the Cochrane Library. Our criteria encompassed studies published between January 2000 and April 2024, with full-text articles written in English. The entirety of our research, comprising clinical study, observational studies, clinical trials, and animal research, reached its conclusion. Inclusion criteria consisted of papers that focused on maternal dietary interventions and their impact on CKM syndrome in offspring. The exclusion criteria were, as follows: (1) papers addressing maternal nutritional status without a focus on specific dietary interventions; (2) studies involving non-food-based interventions; (3) research focusing on offspring outcomes not related to CKM syndrome; and (4) studies limited to fetal outcomes only. Editorials, letters, conference abstracts, and comments were omitted from consideration. Moreover, we scrutinized the reference lists to identify supplementary pertinent sources.

### 3.3. Data Extraction

A search using various keywords across different databases was conducted and yielded 6979 articles. Following the removal of duplicates, 2213 articles were initially screened for relevance to the topic. An additional 129 articles were obtained from linked research and reference lists. From these combined sources, a total of 2342 studies were screened for inclusion based on the predefined criteria. Through a secondary manual screening process, 72 articles were ultimately selected for inclusion in the present scoping review.

## 4. Conclusions

Currently, the significant public health impact of CKM syndrome and its associated disorders remains a major concern, largely because effective preventive interventions remain lacking [167]. Maternal dietary nutrition, along with various early-life environmental factors, plays a crucial role in determining the future risk of CKM syndrome. In recent years, DOHaD sciences have enhanced our understanding of how maternal diet and fetal programming contribute to the developmental origins of CKM syndrome, highlighting their potential as therapeutic targets for prevention. It is worth noting that dietary interventions are considered effective therapies for combating numerous human diseases and promoting health [1,2]. However, several unresolved questions remain regarding their application in the DOHaD field and clinical practice.

First, it is crucial to gather data on the long-term clinical outcomes related to CKM screening, staging, and therapeutic approaches in the pediatric population. Given that CKM syndrome was only defined in 2023, this information is urgently needed to develop effective strategies for the early identification and prevention of CKM syndrome. Second, while there is growing evidence of the beneficial effects of dietary therapies on human health, their specific impact during pregnancy needs further evaluation. These effects are likely to vary across different human populations and animal models. Third, despite advancements in the availability of various maternal dietary interventions, there has been insufficient exploration of their reprogramming effects on each element of CKM syndrome. Future animal studies should focus on improving study designs by using appropriate animal models, developing robust control measures, establishing standardized dosing protocols, and determining the optimal timing for each dietary intervention.

Understanding the distinct mechanisms by which specific nutrient components, such as micronutrients, macronutrients, and metabolites, influence the developmental programming of CKM syndrome is crucial. There is optimism that personalized maternal diets could potentially prevent CKM syndrome and optimize offspring health outcomes.

## Data Availability

Data are contained within the article.
